# New Anti-Leukemic Effect of Carvacrol and Thymol Combination through Synergistic Induction of Different Cell Death Pathways

**DOI:** 10.3390/molecules26020410

**Published:** 2021-01-14

**Authors:** Fatima Bouhtit, Mehdi Najar, Douâa Moussa Agha, Rahma Melki, Mustapha Najimi, Khalid Sadki, Noureddine Boukhatem, Dominique Bron, Nathalie Meuleman, Abdellah Hamal, Laurence Lagneaux, Philippe Lewalle, Makram Merimi

**Affiliations:** 1 Laboratory of Experimental Hematology, Jules Bordet Institute, Université Libre de Bruxelles, 1000 Brussels, Belgium; bouhtitfatima@gmail.com (F.B.); douaa.moussa@gmail.com (D.M.A.); dominique.bron@bordet.be (D.B.); nathalie.meuleman@bordet.be (N.M.); philippe.lewalle@bordet.be (P.L.); 2 Genetics and Immune Cell Therapy Unit, LBBES Laboratory, Faculty of Sciences, University Mohammed Premier, Oujda 60000, Morocco; mnajar@ulb.ac.be (M.N.); r.melki@ump.ac.ma (R.M.); n.boukhatem@ump.ma (N.B.); abdhamal@gmail.com (A.H.); 3 Osteoarthritis Research Unit, Department of Medicine, University of Montreal Hospital Research Center (CRCHUM), University of Montreal, Montreal, QC H2X 0A9, Canada; 4 Laboratory of Pediatric Hepatology and Cell Therapy, Institut de Recherche Expérimentale et Clinique (IREC), Université Catholique de Louvain, 1200 Brussels, Belgium; mustapha.najimi@uclouvain.be; 5 Laboratory of Human Pathologies Biology, Faculty of Sciences, Mohammed V University, Rabat, Agdal-Rabat 10090, Morocco; ksadki1@yahoo.fr; 6 Laboratory of Clinical Cell Therapy, Jules Bordet Institute, Université Libre de Bruxelles, 1070 Brussels, Belgium; laurence.lagneaux@bordet.be

**Keywords:** AML-cancer therapy, drug combination-synergy, bioactive molecules, cell death, apoptotic and non-apoptotic pathways

## Abstract

Acute myeloid leukemia (AML) is a cancer of the myeloid lineage of blood cells, and treatment for AML is lengthy and can be very expensive. Medicinal plants and their bioactive molecules are potential candidates for improving human health. In this work, we studied the effect of *Ptychotis verticillata* (PV) essential oil and its derivatives, carvacrol and thymol, in AML cell lines. We demonstrated that a combination of carvacrol and thymol induced tumor cell death with low toxicity on normal cells. Mechanistically, we highlighted that different molecular pathways, including apoptosis, oxidative, reticular stress, autophagy, and necrosis, are implicated in this potential synergistic effect. Using quantitative RT-PCR, Western blotting, and apoptosis inhibitors, we showed that cell death induced by the carvacrol and thymol combination is caspase-dependent in the HL60 cell line and caspase-independent in the other cell lines tested. Further investigations should focus on improving the manufacturing of these compounds and understanding their anti-tumoral mechanisms of action. These efforts will lead to an increase in the efficiency of the oncotherapy strategy regarding AML.

## 1. Introduction

**Figure 1:**
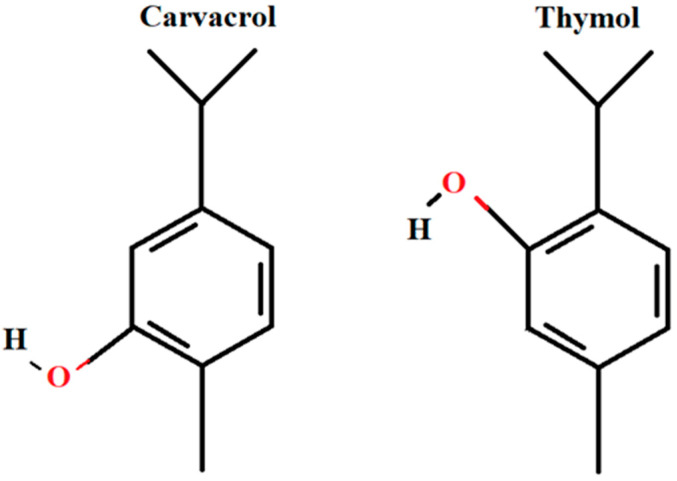
Chemical structure of carvacrol and thymol.

Acute myeloid leukemia (AML) is a heterogeneous hematopoietic stem cell disease that may be cured in some patients with intensive chemotherapy and/or allogeneic stem cell transplantation (allo-SCT) [1]. Despite the approval of new targeted treatments, including FMS-like tyrosine kinase 3 (FLT3) or antiapoptotic B-cell lymphoma 2 (BCL-2) protein inhibitors, up to 50% of AML patients will eventually relapse or die. The major reasons that may explain why patients are not cured are the inability of the treatment to eliminate all cancerous cells and drug-related toxicity to different organs [2]. Cell death evasion mechanisms are a hallmark of tumor resistance to chemotherapy [1]. The major challenge of new anticancer therapeutic solutions is to reactivate cell death programs in cancer cells while having low toxicity to normal cells. An increasing number of studies have shown the beneficial effects of different medicinal plant extracts. Accumulating evidence shows that medicinal plant compounds exert damaging effects on cancer cells. Bioactive molecules are capable of killing cancerous cells through various molecular mechanisms, including intrinsic or extrinsic caspase-dependent apoptosis, autophagy, and necroptosis [1]. *Ptychotis verticillata* (PV) is an endemic Maghrebian plant used in traditional medicine for the treatment of fever, muscle spasms, urinary infections, diabetes, and hypotension [3,4]. The essential oil (EO) of PV contains several compounds, mostly phenolic components (48.0%) with carvacrol (44.6%) and thymol (3.4%) as the main compounds [5]. The chemical structure of carvacrol and thymol is shown in Figure 1 using Microsoft paint software (Microsoft, Corp., Redmond, WA, USA). Few works have been devoted to this plant, and they have only focused on its antimicrobial and antidiabetic effects. Thus, no study has been published to show the anticancer effect of *Ptychotis verticillata* (PV) essential oil (EO) or its cytotoxic effect on normal cells. Recently, we have demonstrated that EO of PV and its constituents, carvacrol and thymol, have shown beneficial effects on different biological features of mesenchymal stromal cell (MSC) such as stimulating proliferation and preventing cellular cytotoxicity [6].

The novelty and originality of our work is to assess the anticancer effect of PV-derived EO and the combination of its two main components on different AML cell lines. We have also evaluated the potential synergistic effect of carvacrol and thymol combination regarding leukemic cell death. Drug combinations are a well-established form of cancer treatment [7]. Administering more than one drug can provide many benefits: higher efficacy, lower toxicity, and at least delayed onset of acquired drug resistance [8,9,10]. Such compounds act on tumors by inducing cell death through different pathways [11,12]. Most of the studies on the cytotoxic effects of plant bioactive components are limited to the effect of single components, while their impact is normally due to the combination of multiple components. At this time, no published work has studied the effect of the combination of these two compounds on cancer cell death induction or the molecular mechanisms associated with it. Our results open the door to the establishment of new anticancer therapeutic strategies for treating AML by focusing on the relevance of the combination of carvacrol and thymol in inducing a potential synergistic cell death of cancer cell lines.

## 2. Results

### 2.1. Cytotoxic Effects of Ptychotis Verticillata (PV) Extract Oil on Myeloid Leukemic Cell Lines

**Figure 2:**
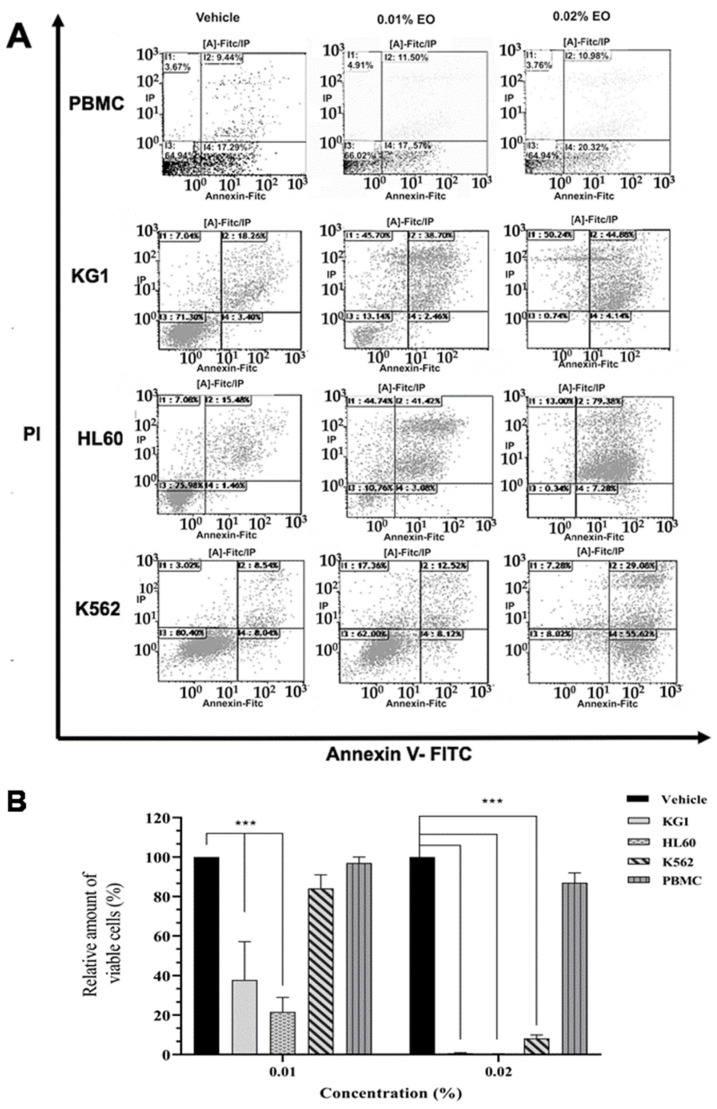
(**A**,**B**) Cell cytotoxicity of *Ptychotis verticillata* Essential oil (PV EO) on myeloïd leukemic cell lines and peripheral blood mononuclear cells (PBMCs) from healthy donors. (**A**): Flow cytometric analysis of cell death with Annexin V-FITC/PI double staining after 24 h treatment of KG1, HL60, and K562 cell lines with increasing concentrations of essential oil. Data were analyzed by Kaluza software 2.1. (**B**): The quantification of myeloïd leukemic cell lines viability compared to PBMCs from a healthy donor after their treatment with the same concentrations of PV EO (0.01% and 0.02%). Data represent the mean ± SEM of three independent experiments relative to vehicle. The levels of statistical significance were *p* < 0.001 (***).

First, we treated the leukemic cell lines K562, HL60, and KG1 with different concentrations of the PV EO extract. These cell lines were selected since they partially reflect the molecular heterogeneity of myeloïd leukemia. As shown in Figure 2, the EO of PV is capable of inducing cell death after 24 h of treatment at a concentration of 0.01% in both HL60 and KG1 cell lines (Figure 2A,B). Although less sensitive at this concentration, most K562 cells are killed using a concentration of 0.02%, as shown in Figure 2A,B. The K562 cell death induced by EO is associated with a high level of annexin+/PI-cells, suggesting the implication of apoptosis pathways. Interestingly, these two concentrations (0.01% and 0.02%) are not toxic to normal cells (Figure 2B). The treatment of PBMCs isolated from the peripheral blood of healthy donors with different concentrations of HE was toxic only at very high concentrations (0.04%) (data not shown). These results show that PV EO induces cell death in HL60, KG1, and K562 at concentrations that are not toxic to normal cells.

### 2.2. Potential Synergistic Cell Death Induction by Thymol and Carvacrol on Myeloïd Leukemic Cell Lines

**Figure 3:**
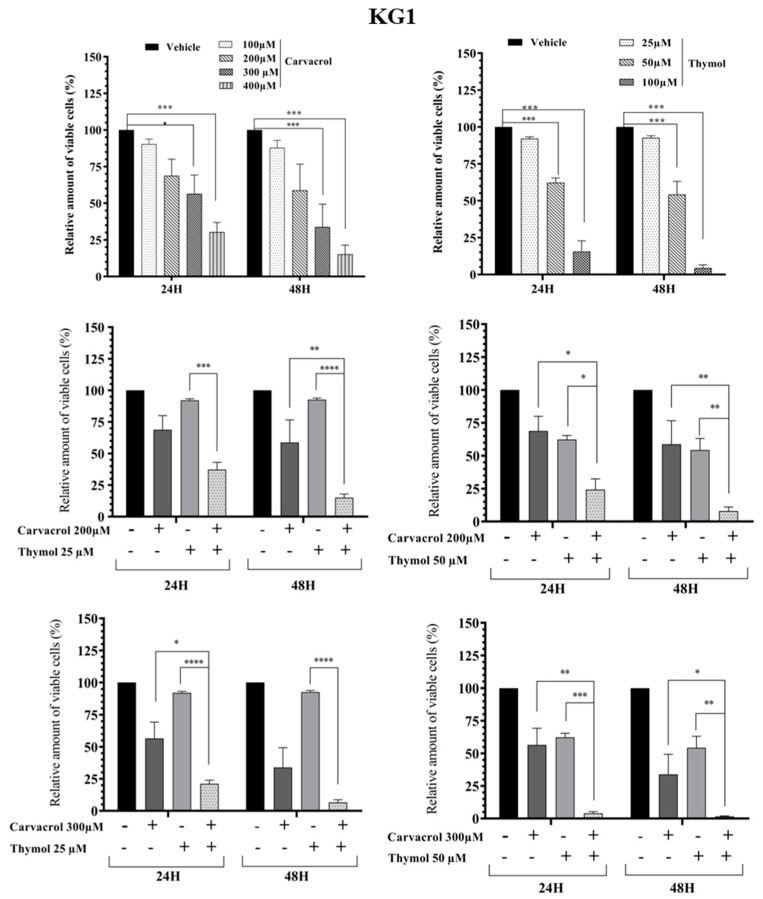
Impact of carvacrol, thymol, and their combination on the viability of the KG1 myeloid leukemic cells. Flow cytometric analysis of cell death with Annexin V-FITC/PI double staining after 24 h and 48 h treatment with different concentrations of carvacrol and thymol (individually or in combination). Data represent the mean ± SEM of three independent experiments relative to vehicle. The levels of statistical significance were *p* < 0.05 (*), *p* < 0.01 (**), *p* < 0.001 (***), and *p* < 0.0001 (****).

**Figure 4:**
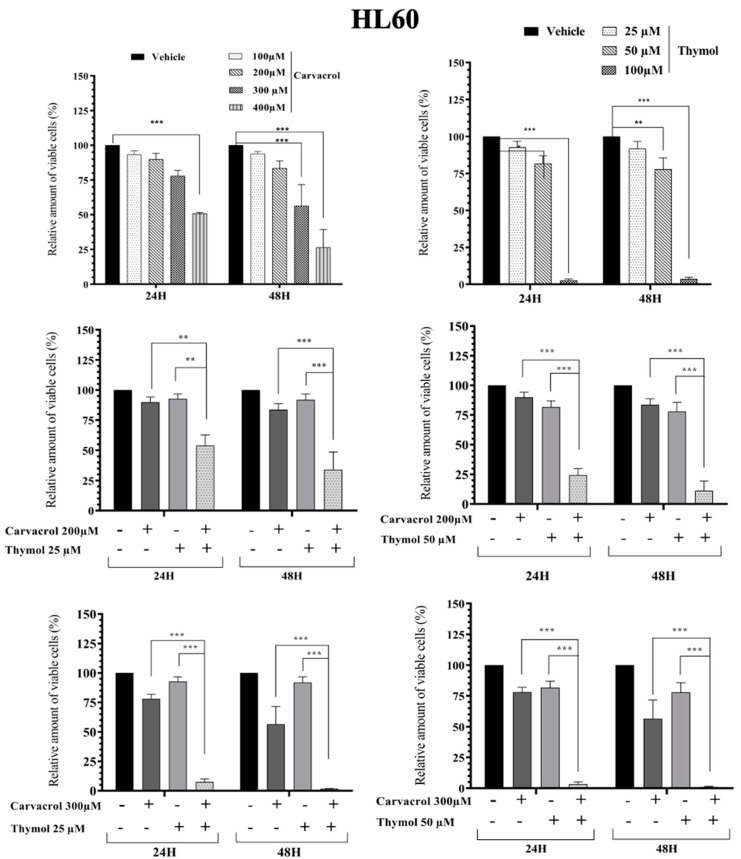
Impact of carvacrol, thymol, and their combination on the viability of the HL60 myeloid leukemic cells. Flow cytometric analysis of cell death with Annexin V-FITC/PI double staining after 24 h and 48 h treatment with different concentrations of carvacrol and thymol (individually or in combination). Data represent the mean ± SEM of three independent experiments relative to vehicle. The levels of statistical significance were set at *p* < 0.01 (**) *p* < 0.001 (***).

**Figure 5:**
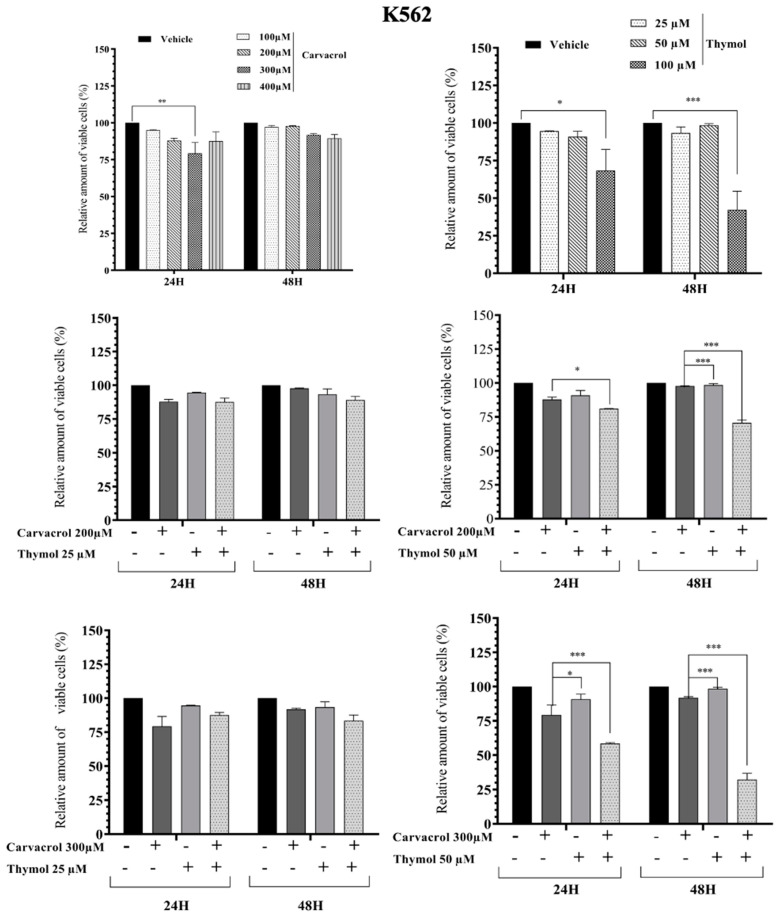
Impact of carvacrol, thymol, and their combination on the viability of the K562 myeloid leukemic cells. Flow cytometric analysis of cell death with AnnexinV-FITC/PI double staining after 24 h and 48 h treatment with different concentrations of carvacrol and thymol (individually or in combination). Data represent the mean ± SEM of three independent experiments relative to vehicle. The levels of statistical significance were set at *p* < 0.05 (*), *p* < 0.01 (**) and *p* < 0.001 (***).

**Figure 6:**
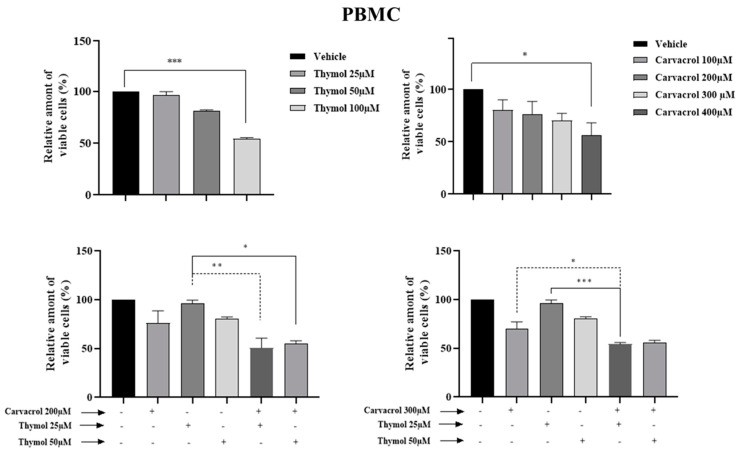
Impact of carvacrol, thymol, and their combination on the viability of the peripheral blood mononuclerar cells (PBMCs) from healthy donors. Flow cytometric analysis of cell death with AnnexinV-FITC/PI double staining after two 48 h treatments with different concentrations of carvacrol and thymol (individually or in combination). Data were analyzed by Kaluza software 2.1. Data represent the mean ± SEM of three independent experiments relative to vehicle. The levels of statistical significance were set at *p* < 0.05 (*), *p* < 0.01 (**) and *p* < 0.001 (***).

As known, PV EO is mainly composed of phenolic compounds (48.0%) with carvacrol (44.6%) and thymol (3.4%) as the majors active compounds [5]. In this part of the work, we studied the combined effect of carvacrol (car) and thymol (thy) on myeloid leukemic cell death. We first tested different concentrations of thymol and carvacrol to determine the concentrations that could be used to study their potential synergistic effect. As shown in Figure 3, KG1 cell lines are very sensitive to carvacrol at 300 µM compared to the HL60 line (Figure 4), while the K562 line (Figure 5) shows resistance after 48 h treatment at 400 µM carvacrol. For thymol, at 50 µM, KG1 cells are sensitive compared to the other two lines. At 100 µM, thymol can induce complete cell death of KG1 and HL60 cells (Figure 3 and Figure 4), whereas around 50% of K562 cells resist cell death after 48 h of treatment (Figure 5). Based on these results, we tested two different concentrations of thymol (25 µM and 50 µM) and carvacrol (200 µM and 300 µM) to study their potential synergistic effect. As shown in Figure 3, for the KG1 cell line, we observed the potential synergistic effect of thymol and carvacrol at the different combinations of drug concentrations after both 24 and 48 h of treatment. For the HL60 line (Figure 4), the potential synergistic effect is more pronounced for the 200 µM car/50 µM thy and 300 μM car/50 µM thy combinations than in other combinations. Indeed, for this cell line, more than 80% of cells remained alive after an individual treatment of the two drugs, but their combination eliminated almost all the cancerous cells. Interestingly, for the K562 cell line (Figure 5) that was less sensitive to individual concentrations of carvacrol and thymol (more than 90% viability after 48 h of treatment with 300 µM carvacrol or 50 µM thymol), the combination of the two drugs was able to kill 70% of the malignant cells. Then, we tested the toxicity of these two drugs on PBMCs from healthy donors. As shown in Figure 6, carvacrol and thymol show toxicity at very high concentrations (carvacrol 400 µM and thymol 100 µM), but more than 50% of PBMCs remain viable after 48 h of treatment. Indeed, when combining the two drugs, more than 50% of the normal cells remain alive even at concentrations that are able to eliminate all KG1 and HL60 cells and more than 70% of K562-resistant cells (Figure 3, Figure 4 and Figure 5). These results suggest that much of the observed antileukemic effect of PV EO could be due to the presence of thymol and carvacrol and that the combination of the two drugs can eliminate even the K562-resistant cells, while presenting less toxicity to normal cells.

### 2.3. Carvacrol- and Thymol Combination-Induced Cell Death Is Associated with Apoptotic, Oxidative, and Cell Stress Pathways (Gene and Protein Levels)

#### 2.3.1. Upregulation of Proapoptotic Gene Expression

**Figure 7:**
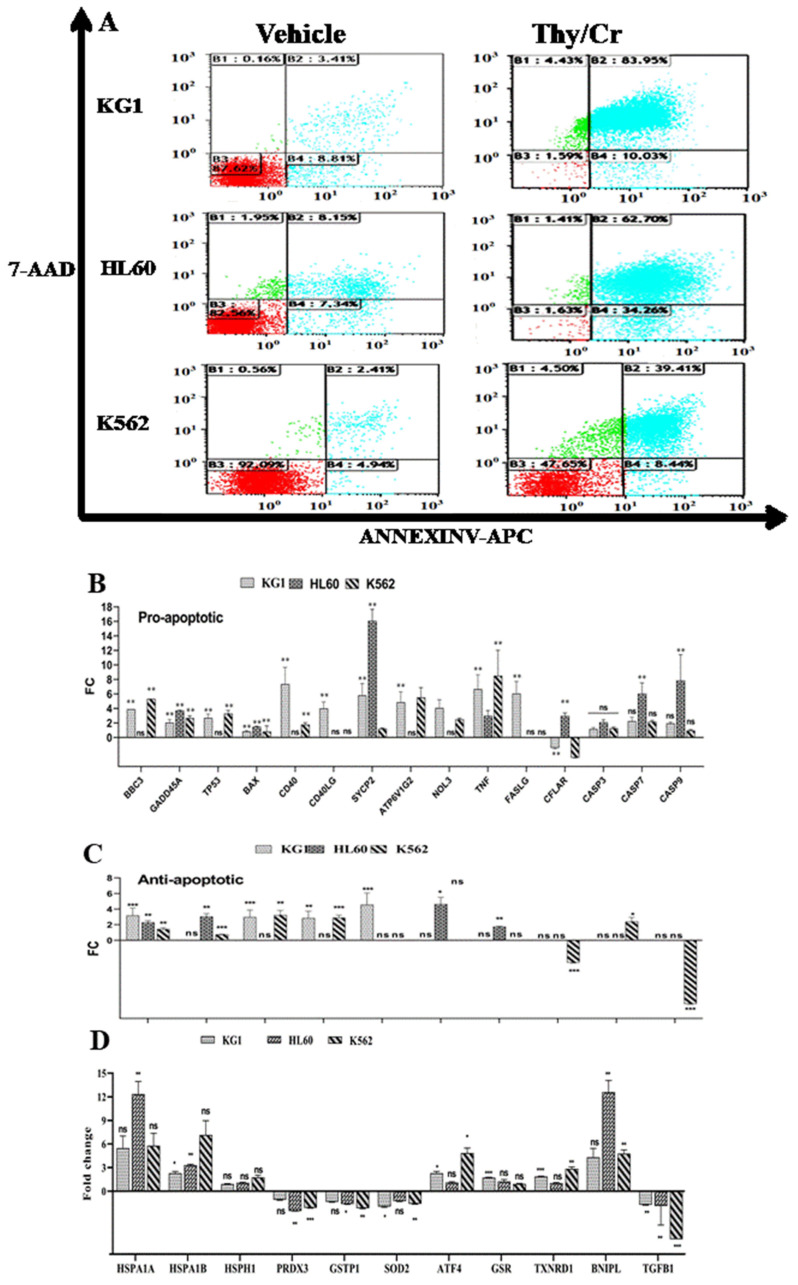
(**A**–**D**) Effect of carvacrol and thymol combination in cell death induction through apoptotic and cell stress pathways. (**A**). Flow cytometric analysis of cell death with Annexin V-APC/7-AAD double staining after 48 h treatment of KG1, HL60, and K562 cell lines with Thy 50µM/Cr 300 µM combination. Data were analyzed by Kaluza software 2.1. (**B**) and (**C**). Relative expression of genes related to apoptotic cell death. (**D**) Relative expression of genes related to cell and oxidative stress pathway. Data represent the mean ± SEM of three independent experiments relative to vehicle. The levels of statistical significance as done by Anova test were set at *p* < 0.05 (*), *p* < 0.01 (**) and *p* < 0.001 (***). ns: no significant.

Different pathways are associated with drug-induced cell death, and it is important to elucidate cell death mechanisms to improve the efficacy of any tested anticancer molecule. Using the allophycocyanin annexin V conjugate (APC–annexin), which is useful for detecting translocated phosphatidylserine (PS), a hallmark of apoptosis, we detected a wide range of annexin+/PI-HL60 (34%), K562 (8%) and KG1 (10%) cells even after 48 h of carvacrol and thymol treatment (Figure 7A). Based on these results, we analyzed the expression of genes that are responsible for apoptotic cell death using a gene expression array from Qiagen. As shown in Figure 7B, Car–thy combination-induced cell death is associated with a significantly upregulated expression of proapoptotic genes, such as BBC3, BNIPL, GADD45A, TP53, BAX, CD40, CD40LG, SYCP2, ATP6V1G2, NOL3, BIRC2, TNF, and FASLG. Some of these genes are significantly upregulated in all three cell lines, while the others are upregulated but statistically significant in only one or two of the cell lines. The BAX gene is overexpressed only in the HL60 line, whereas the caspase 8-like gene (CFLAR) is overexpressed in this cell line and underexpressed in the KG1 and K562 cell lines. The expression of CASP3, CASP7, and CASP9 was elevated but the only significant changes were caspase-9 and caspase-7 in the HL60 cell line. Interestingly, some antiapoptotic genes are also expressed: IGF1R, TRAF2, BIRC3, and XIAP (Figure 7C). This could be explained by a stress response that induces the expression of genes as a protective response to cell death.

#### 2.3.2. Differential Modulation of the Oxidative and Stress Gene Pathways

The expression of certain proapoptotic genes known to be responsible for DNA damage and able to induce reactive oxygen species (ROS) production and cell stress prompted us to analyze the expression of certain genes involved in oxidative and cellular stress. As shown in Figure 7D, cell death induced by the potential synergistic effect is associated with the underexpression of PRDX3, GSTP1, SOD2, and TGFB, which provide antioxidant or ROS production inhibitor function. We also observed the overexpression of TXNRD1, which can serve as an ROS generator, and the overexpression of heat shock proteins and ATF4, which play an important role in reticular stress. These results suggest the involvement of apoptotic and cellular stress pathways in cell death induced by the carvacrol and thymol combination.

#### 2.3.3. Protein Confirmation of Apoptotic and Reticular Stress Signaling Pathway Implication

**Figure 8:**
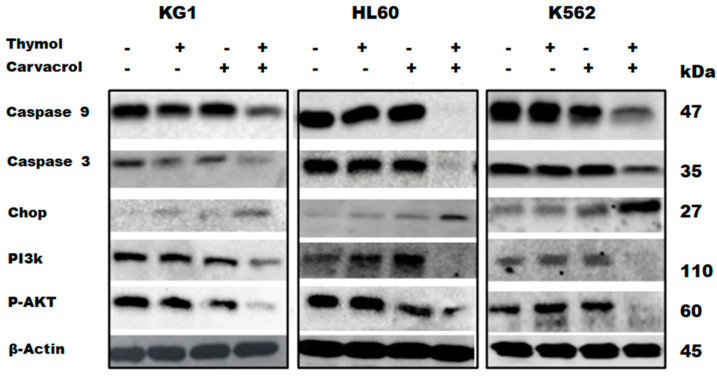
Effect of carvacrol and thymol combination in cell death induction through apoptotic and cell stress pathways. Western blot (WB) of caspase-3, caspase-9, CHOP, PI3k, pAKT, and B actin expression following carvacrol and thymol treatment (individually or in combination). A representative figure of WB results from three independent experiments is presented.

To confirm the results obtained in Figure 7D, the expression of these genes was analyzed by Western blot. As shown in Figure 8, the carvacrol and thymol combination shows a potential synergistic effect by inducing the decrease of procaspase-3 and procaspase-9 expression and the increase of CHOP expression. CHOP is involved in reticular stress, and it is known that ROS can trigger endoplasmic reticulum (ER) stress or vice versa in vivo and in vitro [13]. We also analyzed the protein expression of PI3K and p-AKT to determine how the carvacrol and thymol combination may modulate the antiapoptotic mechanisms of cell death resistance. As previously stated, we observed an upregulation of insulin growth factor 1 receptor (IGF1R) gene expression in Figure 7C. This is important, as a sustained activation of AKT/mTOR in malignant-resistant cells is regulated by the upregulation of the IGF1R [14]. As shown in Figure 8, the potential synergistic effect of carvacrol and thymol in the induction of leukemic cell death is also associated with the decrease of PI3K and p-AKT protein expression in combination-treated cells compared to vehicle and cells treated with carvacrol or thymol separately. These Western blot results confirm that the combination of carvacrol and thymol induces cell death through apoptotic, oxidative, and cell stress pathways. Gene and protein expression profiling indicates that the potential synergistic effect of this combination is associated with both cell death and antiproliferative signaling pathways.

### 2.4. Carvacrol- and Thymol Combination-Induced Potential Synergistic Cell Death Is Associated with Nonapoptotic Pathways

#### 2.4.1. Dependence on Caspases Activation and ROS Generation Pathways

**Figure 9:**
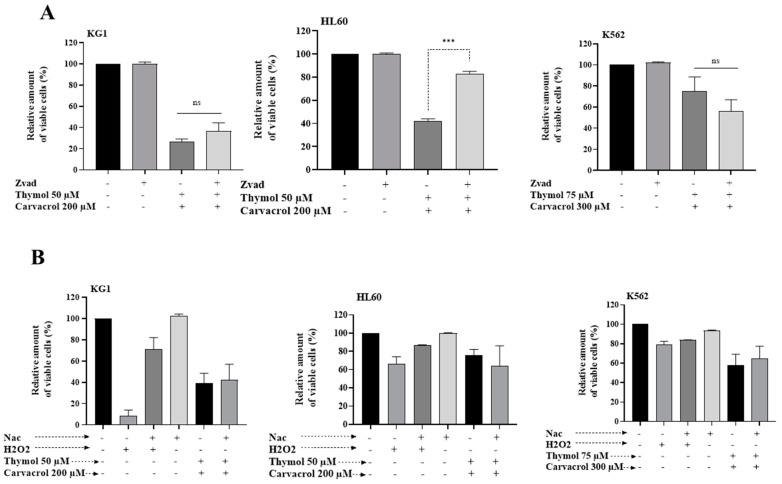
(**A**,**B**) Effect of carvacrol and thymol combination in cell death induction through non-apoptotic pathways. Flow cytometric analysis of cell death with Annexin V-APC/7-AAD double staining after 48 h treatment of KG1 and HL60 cell lines with the combination of 50 µM Thy/200 µM Cr and K562 cell line with 75 µM Thy/300 µM Cr, in the presence of the pan caspase inhibitor Z-VAD (**A**) or with the ROS scavenger NAC (**B**). Data were analyzed by Kaluza software 2.1. The levels of statistical significance as done by Anova test were set at *p* < 0.001 (***). ns: no significant.

To determine if alternative molecular mechanisms might be involved in the cell death induced by the combination of carvacrol and thymol, we first studied the dependence of this induced cell death on the caspase activation pathway and on ROS generation. The cells were treated with Z-VAD-FMK, a pan caspase inhibitor, 1 h before treatment with both drugs. As shown in Figure 9A, only the HL60 line had a dependence on the caspase pathway. Indeed, in this cell line, pretreatment with a caspase inhibitor abrogated the car–thy-induced cell death. The other two cell lines showed no significant change in the cell death level following the inhibition of caspases. In addition, the use of the ROS scavenger *N*-acetyl-l-cysteine (NAC) had no effect on car–thy-induced cell death in the three cell lines (Figure 9B). These results suggest that the ability of the combination of the two drugs to induce other cell death pathways is independent of caspase activation and ROS generation.

#### 2.4.2. Implication of Autophagy and Necrosis Pathways

**Figure 10:**
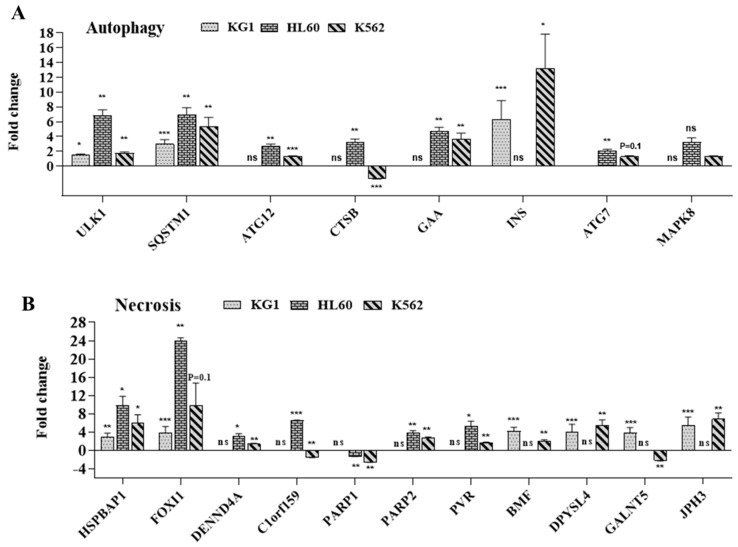
(**A**,**B**) Effect of carvacrol and thymol combination in cell death induction through non-apoptotic pathways. Relative expression of genes related to autophagy (**A**) and necrosis (**B**) cell death pathways. The levels of statistical significance as done by Anova test were set at *p* < 0.05 (*), *p* < 0.01 (**) and *p* < 0.001 (***). ns: no significant.

Cell death is often regulated by multiple molecular pathways and mechanisms, including apoptosis, autophagy, and necrosis. The molecular network underlying these processes is often intertwined, and one pathway can dynamically shift to another due to the role of certain genes, such as TP53, TNF, and ATF4, in different cell death pathways. Therefore, we analyzed the expression of genes involved in autophagy and necrosis in the three cell lines treated with the combination of carvacrol and thymol. As shown in Figure 10, car–thy-induced cell death is associated with the overexpression of genes implicated in autophagy (Figure 10A), including ULK1, SQSTM1, ATG12, GAA, INS, MAP1LC3A, and TP53, and necrosis (Figure 10B), such as HSPBAP1, FOXI1, DENND4A, C1orf159, PARP1, PARP2, PVR, ATP6V1G2, BMF, DPYSL4, and GALNT5. A few of these genes are significantly upregulated in the three cell lines, while others are upregulated but statistically significant in only two cell lines. Together, these results showed that car–thy-induced cell death in leukemic cells, except in HL60 cells, is associated with non-apoptotic and caspase-independent pathways in leukemic cell lines. The induced cell death in the HL60 cell line is caspase-dependent, ROS production-independent, and associated with non-apoptotic pathways. In conclusion, the combination of thymol and carvacrol has a potential synergistic effect on myeloid leukemia cell death induction by apoptotic and non-apoptotic pathways.

## 3. Discussion

Cancer is the second leading cause of death worldwide. Despite intensive research on the treatment of this disease, the molecules on the market face different obstacles. The high cost of treatment, the molecular heterogeneity of cancer cells, and the development of drug resistance are the major challenges to be overcome. Acute myeloid leukemia (AML) is a cancer of the myeloid lineage of blood cells, with high molecular and genetic heterogeneity. The treatment is still expensive and may be associated with several side effects [15]. Despite the development of stem cell transplantation, more than 50% of patients relapse. Herbal remedies and their bioactive molecules are potential candidates for improving human health. Natural compounds from certain plants exhibit antiproliferative properties and cell death induction in various cancers through different pathways, including apoptosis, autophagy, and necroptosis [1]. The ability of these compounds to eliminate tumor cells both ex vivo and in vivo suggests their potential as new anticancer therapeutics. *Ptychotis verticillata* (PV) is a plant used in traditional medicine to treat diabetes, hypertension, and other diseases. *Ptychotis verticillata* (PV) is a plant known by the vernacular name “Nûnkha” or “Nanunkha”. It belongs to the Apiaceae or Umbelliferae family, and it grows spontaneously in North Africa, from Morocco to Libya. It is also found throughout the Mediterranean basin. PV is an annual plant with a slender taproot, rod erect, striated, and slender, with many spreading branches from 10 to 40 cm, without rosette of basal leaves. In Morocco, Nûnkha is an aromatic and medicinal plant mainly found in the eastern region [5]. PV is widely used in traditional medicine as an infusion to treat headache, fever, flu, diarrhea, diabetes, and hypertension [16]. The use of individual compounds or in combination rather than the essential oil is a relevant question to be investigated. In fact, additional information regarding the composition of the PV is needed. Analysis of the chemical composition of PV essential oil from Morocco was carried out using GC and GC-mass spectrometry. The oil was dominated by phenolic compounds (48.0%) with carvacrol (44.6%) and thymol (3.4%) as the main compounds. Plant phenolics constitute one of the major groups of components that act as primary antioxidant free radical terminators [5]. The amounts of total phenolics and flavonoids in the solvent extracts (diethyl ether and ethyl acetate) were determined spectrometrically. Furthermore, the antioxidant activities of the essential oil and extracts were determined using a DPPH test system. The DPPH scavenging activity of extracts increased in the order ethyl acetate > ascorbic acid > diethyl ether > essential oil. The antimicrobial activity of the essential oil of *P. verticillata* (EOPV) and its different fractions has shown a higher inhibitory effect on different bacterial strains [17]. Thus, PV is a potential rich source of antimicrobial agents against many microorganisms and could be used as an antibiotic or alternative. Although some reports have shown the anticancer effect of thymol and carvacrol, no study has yet evaluated the combination of these two molecules against malignant cells. Most importantly, looking for a potential synergistic action of these active compounds is required for an efficient treatment.

The originality and novelty of our work is to demonstrate the anticancer effect of PV EO and the potential synergistic combination of its two components, carvacrol and thymol, against myeloid leukemic cell lines. This is the first time that a study has investigated the anticancer effect of PV EO or its cytotoxic effect on normal cells. Our results showed that the 0.01% and 0.02% concentrations of PV EO, which are nontoxic to normal cells, can eradicate all leukemic cells, suggesting that the oil constituents play a dual role in cell viability. At similar concentrations, they can kill cancerous cells, and on the other hand, they are able to protect normal cells from drug toxicity. Recently, we showed that Ptychotis verticillate EO, carvacrol, and thymol have several positive effects on MSCs, which are known to be an important immunotherapeutic cell product [18]. By preserving their morphology, sustaining their viability, promoting their proliferation, and protecting them from cytotoxicity and oxidative stress, PV appears to be a promising natural medicinal product for strengthening the therapeutic effects of MSCs [6]. Several studies have shown that carvacrol or thymol may individually induce, in vitro and in vivo (animal models), cancer cell death through the deregulation of different genes involved in cell proliferation and the apoptosis signaling pathway [11,12]. For leukemic cells, previous studies have shown that both carvacrol and thymol individually showed cytotoxic effects on the HL60 cell line by inducing apoptotic cell death [19,20]. However, at this time, no published work addressed the effect of the combination of these two molecules on the induction of cancer cell death. Thus, we aimed to investigate the anticancer activity of the combination of carvacrol and thymol in three different leukemic cell lines that are able to partially represent the molecular heterogeneity of AML [21]. Our results showed a potential synergistic effect of thymol and carvacrol at the different combinations used in the KG1 and HL60 cell lines. Interestingly, for the K562 cell line, which was far less sensitive to individual treatment with carvacrol or thymol, the combination of the two drugs was able to eliminate 70% of K562 cancer cells. The drug concentrations used are toxic to normal cells, but more than 50% of the cells remain alive, even at concentrations that can eliminate all KG1 and HL60 cells and more than 70% of K562-resistant cells. These results show that much of the observed antileukemic effect of PV EO is due to the presence of thymol and carvacrol and that the combination of the two drugs can eliminate even the K562-resistant cells while presenting less toxicity to normal cells. However, the nontoxic effect of EO compared to the combination of carvacrol and thymol (car–thy) in normal cells may be due to the existence of other compounds in EO that protect normal cells from cytotoxicity and oxidative stress. These results prompted us to study the molecular mechanisms involved in this cell death induced by the potential synergistic effect. Our results showed a wide range of annexin+/PI-cells (especially for HL60 cell lines) after carvacrol and thymol treatment associated with the expression of the proapoptotic genes BBC3, GADD45A, TP53, BCL2L11, BAX, CD40, CD40LG, SYCP2, ATP6V1G2, NOL3, TNF, and FASLG. We confirmed by Western blotting that the induction of leukemia cell death is associated with apoptotic pathways, as shown by the strong decrease in procaspase-3 and procaspase-9 in cells treated with the combination of carvacrol and thymol. The GADD45A gene is an indicator of DNA damage and plays a role in drug-induced apoptosis with the involvement of stress-induced MAPK8 [22]. The DNA damage-induced transcription of this gene is mediated by both p53-dependent and p53-independent mechanisms. Our results showed an increase in TP53 and GADD45A expression after car–thy treatment, suggesting that DNA damage and oxidative stress are involved in car–thy-induced cell death. It was shown that p53 promotes the expression BBC3 (also upregulated in our results), leading to increased intracellular ROS levels and apoptosis induction [23]. BBC3-induced apoptosis is directly associated with ROS generation [24]. Our cell and oxidative stress transcriptomic results confirm the involvement of ROS in car–thy-induced cell death. Indeed, the potential synergistic effect is associated with the underexpression of PRDX3, GSTP1, and SOD2, which provide antioxidant or ROS generation inhibitor functions, as well as the overexpression of TXNRD1, which can serve as an ROS generator [25]. Our results also showed downregulation of the TGFB gene, which is known to control ROS production directly or by downregulating antioxidative systems [26]. It has been demonstrated that reactive oxygen species (ROS) production is also increased by TGF-β downregulation, which triggers Akt inactivation in cancer cell death [13]. The failure of leukemic cells to resist this potential synergistic effect may be explained by the upregulation of antiapoptotic genes, such as the insulin growth factor 1 receptor (IGF1R). In cancer-resistant cells, the sustained activation of the AKT/mTOR is regulated by the upregulation of IGF1R [14], which plays a determinate role in promoting resistance to PI3K/AKT/mTOR inhibitors. Our Western blot results confirmed this failure to resist to cell death probably due to a decrease in PI3K and p-AKT expression in combination-treated cells compared to untreated cells and cells treated with carvacrol or thymol alone. Furthermore, the overexpression of the proapoptotic gene BCL211 (BIM) could reinforce this resistance failure. Indeed, Locatelli et al. demonstrated the role of BIM in arsenic trioxide-induced ovarian cancer cell death through the activation of caspase-3 and dephosphorylation of p-AKT [27]. These results suggest that the potential synergistic effect of car–thy can escape the mechanisms of cell death resistance in part by inhibiting cell proliferation through the PI3K/AKT signaling pathway. In addition, accumulating evidence suggests the interrelation of ROS and endoplasmic reticulum stress (ER) with redox signaling mediators [28]. ROS can trigger endoplasmic reticulum (ER) stress or vice versa in vivo and in vitro [13]. Under prolonged and severe ER stress conditions, the unfolded protein response (UPR) can become cytotoxic (including apoptosis) rather than cytoprotective. Interestingly, in our results, car–thy-induced cell death is associated with the upregulation of ATF4, which is one of the UPR-dependent signaling proteins. C/EBP-homologous protein (CHOP) is another important component of the endoplasmic reticulum (ER) stress response. CHOP knockdown significantly attenuated ER stress-induced apoptosis in hepatocarcinoma cells [29]. CHOP and ATF4 cooperate to induce cell death, although how this is achieved is still not completely understood [30]. Our Western blot results showed an increase in CHOP levels in cells treated with the drug combination and confirmed the implication of ER stress in car–thy-induced leukemic cell death. Together, these results show that the association of carvacrol and thymol induces cell death through apoptotic, oxidative, and reticular stress pathways and suggest that all these mechanisms collaborate to escape cancer cell death resistance. Human cancer treatment strategies ultimately rest on the ability of cancer therapeutics to induce tumor-specific cell death. However, cancer cells, because of their molecular heterogeneity, may present genetic or epigenetic modifications of certain pathways that render them unresponsive to treatment [31]. The car–thy-induced cell death transcriptomic profile shows the expression of certain antiapoptotic genes as well as the expression of antioxidant genes. In addition, the expression profile of certain genes following the cell death induced by the potential synergistic effect of the two drugs depends on the molecular heterogeneity of the different cell lines; for example, the BBC3, TP53, CD40, ATP6V1G2, and NOL3 genes are significantly upregulated following drug treatment in KG1 and K562 cells, whereas BAX and CFLAR (caspase-8-like) genes are only upregulated in HL60 cells. Our studies of cell death dependence on the caspase pathway and ROS generation showed that cell death induced by the potential synergistic effect of car–thy is independent of both pathways in all the cell lines treated, except for HL60, which shows a strong dependence on the caspase pathway. These results could partly explain the overexpression of Bax and CFLAR in this cell line following treatment and the inability of HL60 to express certain genes that may play a role in both apoptotic and nonapoptotic cell death pathways, such as CD40, TNF, TP53, ATF4, and ATP6V1G2 [32,33,34,35]. Cell death is often regulated by multiple molecular pathways and mechanisms, including apoptosis, autophagy, and necroptosis. The molecular networks driving these different processes are linked, and one pathway may dynamically evolve to another, particularly during cell treatment with various drugs [36]. To determine the non-apoptotic pathways induced by the car–thy combination, we analyzed the profile of a set of genes involved in autophagy and necrosis. The results showed the overexpression of a set of genes that are involved in both pathways. The comparison of the different lines according to apoptosis dependence did not show a large demarcation between the HL60 line and the other cell lines. However, we observed a significant increase in the expression of some genes, including necrosis genes BMF, DPYSL, and JPH3 and autophagic genes INS and TP53, in only the KG1 and K562 cell lines, in which car–thy-induced cell death is caspase independent. It seems that the inability of car–thy to induce the expression of certain genes that allow the switch between the different pathways in HL60 is one of the causes of the dependence of this line on the caspase pathway. However, this dependence does not seem to be absolute. An extension in car–thy treatment time restores cell death even in the presence of caspase inhibitor. This could be explained by the induction of the expression of a dozen autophagy and necrosis genes in this cell line in response to car–thy.

Collectively, these results show that the combination of carvacrol and thymol induce a potential synergistic cell-death effect against acute leukemia cells by activating apoptotic, oxidative, and reticular stress pathways. In addition, the activation of non-apoptotic pathways such as autophagy and necrosis may participate in this induced cell death. Therefore, we determined that several molecular pathways are modulated both at gene and protein levels. These results may contribute to the efforts in developing a safe and efficient anti-AML strategy based on thymol and carvacrol combination. Both of these molecules should be therapeutically evaluated using mouse models and ultimately clinical trials. For instance, there are few clinical data regarding these molecules. In this context, carvacrol has been approved as a food additive by the Food and Drug Administration (FDA) and added in the list of chemical flavorings by the Council of Europe. It is known that carvacrol has several pharmacological properties including antioxidant, anti-inflammatory, immunomodulatory, antitumor, and antimicrobial effects [12]. A clinical study that was conducted to assess the effect of carvacrol on respiratory symptoms in the veterans exposed to sulfur mustard (SM) showed that two months of treatment with carvacrol reduced inflammatory cytokine and chemokine but increased anti-inflammatory cytokines. Such treatment also improved respiratory symptom and forced expiratory volume—one value second in SM-exposed patients [37]. Another Phase II clinical trial was conducted in 2018 to examine the preventive effect of carvacrol in asthmatic patients during a 2-month treatment period. Pulmonary function tests in these carvacrol-received patients were increased, and respiratory symptoms and inflammatory parameters were reduced, which indicates its therapeutic effect on asthma [38]. In contrast, the vast majority of the published studies regarding thymol are in vitro or preclinical data. In all of these studies, thymol pre- and co-treatment in rats appear devoid of any deleterious effects, which is suggestive of its safety [11]. These studies recommend performing clinical trials to determine the exact dosage of thymol against cancer disease in humans. Interestingly, the effect of feeding a carvacrol–thymol blend supplemented diet was evaluated in piglets in which weaning induced intestinal oxidative stress and dysfunction of the intestinal barrier [39]. The results indicated that dietary supplementation with carvacrol–thymol decreased the intestinal oxidative stress and influenced selected microbial populations without changing the biomarkers of intestinal barrier in weaning piglets. Therefore, it is recommended that experiments using mouse models and ultimately clinical trials are implemented to evaluate the combination of carvacrol and thymol for the management of AML. The amelioration of compound manufacturing and characterization is also an important feature for developing a safe, quality, and efficient natural therapeutic product. Comprehensive therapeutic and toxicological studies using the carvacrol–thymol combination should be conducted using animal models (e.g., leukemic mouse models) followed by well-designed clinical studies. Collectively, increasing the knowledge regarding the potential synergistic anti-tumoral mechanisms of action of the thymol and carvacrol combination will provide new onco-therapeutic perspectives for the management of AML.

## 4. Materials and Methods

### 4.1. Plant Material and Essential Oil Isolation

The aerial parts of *P. verticillata* were collected during the flowering season in May 2018 and 2019 (full bloom) from Morocco. Voucher specimens were deposited in the herbarium of Mohamed 1st University, Oujda, Morocco. Fresh vegetal material was water distillated (3 h) using a Clevenger-type apparatus according to the method recommended in the European Pharmacopoeia (Council of Europe, 1996). The essential oil yields were 2% (*w*/*w*). The oils were dried over anhydrous sodium sulfate and then stored in sealed glass vials at ambient temperature prior to analysis.

### 4.2. Chemicals Reagents

All chemicals, unless otherwise stated, were of the highest quality and were used as supplied. Carvacrol (99.9%), thymol (98.5%), dichlorofluorescin diacetate (DCFH-DA), *N*-acetyl-l-cysteine (NAC), and trypan blue were purchased from Sigma-Aldrich (St. Louis, MO, USA). RPMI 1640 culture medium with l-glutamine, fetal bovine serum (FBS), penicillin–streptomycin, Hank’s Buffered Saline Solution (HBSS), and phosphate-buffered Saline EDTA (PBS) were purchased from Lonza (Basel, Switzerland). Dimethylsulfoxide (DMSO) was obtained from Molecular Probes (Eugene, OR, USA). Annexin-V-FITC, propidium iodide (PI), and Z-VAD-FMK were obtained from BD Pharmingen (San Diego, CA, USA). Caspase-3, caspase-9, Chop, p-AKT, PI3K, and β-actin antibodies were obtained from Cell Signaling (Danvers, MA, USA).

### 4.3. Cell Lines, Culture Conditions, and Drug Treatment

Human AML cell lines KG1, HL60, and K562 were purchased from Sigma-Aldrich. All cell lines were grown in complete culture medium containing 90% RMPI-1640 culture medium supplemented with 10% heat-inactivated fetal bovine serum, 2 mM l-glutamine, and 50 U of antibiotic (penicillin and streptomycin). Cells lines were incubated at 37 °C with 100% humidity and 5% CO_2_. The cells were permanently maintained in the exponential phase of cell growth; cells were plated at an adequate density and checked every 2 days. Blood samples are collected from voluntary donors. Volunteer informed consent was approved by the Ethical Committee of Institut Jules Bordet. Peripheral blood mononuclear cells (PBMCs) were purified by Ficoll gradient separation using Histopaque-1077 from Sigma. In brief, blood samples were collected in sterile tubes containing ACDA; then, samples were diluted in HBSS containing 10% ACDA. Two parts sample were carefully added to one-part Ficoll, and cell separation was performed by centrifugation at 2000× *g* for 20 min. After the centrifugation steps, mononuclear cells were identified as the opaque cellular layer, while erythrocytes and granulocytes were pelleted to the bottom. Then, the cellular layer of PBMCs was carefully transferred to new sterile tubes. Finally, PBMCs were washed twice with PBS-EDTA containing 2% SSPP (stable pasteurized solution of plasma proteins), and the cell pellets were resuspended in complete culture medium. Cell viability for the isolated peripheral blood mononuclear cell (PBMC) was assessed using trypan blue dye exclusion and was always >95%. Essential oil, thymol, and carvacrol were dissolved in ethanol or DMSO to obtain a stock solution of 0.1% for EO, 1 mM for thymol, and 10 mM for carvacrol. The final DMSO concentration for cell treatments was < 0.1%. Unless otherwise mentioned, cells were seeded (0.8 × 10^6^ cells/mL) and treated at 50% confluency with EO, thymol, and carvacrol at the desired concentration for 24 or 48 h. For most studies, AML cells and PBMCs were treated with different concentrations of carvacrol (100, 200, 300, and 400 µM) and thymol (25, 50, 75, and 100 µM).

### 4.4. Trypan Blue Assay

A trypan blue dye exclusion assay was used to evaluate the efficacy of separate or combined thymol and carvacrol treatments in comparison to control treatment. Cells were seeded in 12-well plates at a density of 0.8 × 10^6^ cells/mL. At 50% confluency, cells were treated in triplicate with different thymol and carvacrol concentrations as well as their combinations. Then, cells were mixed with equal volumes of dye and mounted on a hemocytometer for counting under the microscope. The results are expressed as percentage of viable cells relative to the control. The following formula was used to calculate the percentage of viable cells: %viability = [number of viable cells (treatment)/number of viable cells (control)] × 100.

### 4.5. Cell Viability Assay

The MTS (3-(4,5-dimethylthiazol-2-yl)-5-(3-carboxymethoxyphenyl)-2-(4-sulfophenyl)-2*H*-tetrazolium) assay was performed to assess the impact of the drugs on cell growth using the CellTiter 96^®^ AQueous One Solution following the manufacturer’s instructions from Promega Benelux (Leiden, The Netherlands). Briefly, cell plating was performed in 96-well plates at a density of 40,000 cells per well in 100 µL complete culture medium in the presence or absence of the indicated drugs. After 48 h of incubation, 20 µL of MTS was added to each well and incubated again for up to 3 h, and the absorbance was measured by using an EX Microplate Photometer (Thermo Scientific, Waltham, MA, USA) at a wavelength of 490 nm. Growth inhibition was calculated by comparing the absorbance values of drug-treated cells to that of untreated controls, which was set at 100%; the IC_50_ values were calculated from sigmoidal dose response curves using Prism 8.2 (GraphPad, San Diego, CA, USA).

### 4.6. Cell Death Assay

Cells were seeded in 12-well plates at a density of 0.8 × 10^6^ cells/mL. After incubation with different drugs, cells were harvested and washed twice with PBS-EDTA. Cell death was determined using an Annexin-V-FITC/PI (propidium iodide)-based apoptosis detection kit from BD Pharmingen kit (556547) following the manufacturer’s instructions. After staining, the cell death of PBMCs was evaluated by flow cytometry (Navios-Beckman Coulter), and the data generated were analyzed by Kaluza software 2.1 (Beckman Coulter, Brea, CA, USA). We have considered the value of the DMSO (vehicle) effect as 100% viable cells for each cells type. We calculated the drug effect with subtraction of the vehicle effect for each cells type.

### 4.7. RNA Isolation and Q-PCR Analysis

Cells were seeded in 6-well plates at a density of 0.8 × 10^6^ cells /mL; treated cells were harvested and washed twice with cold PBS, and then, total cellular RNA was extracted by using the Tripure Isolation Reagent following the manufacturer’s instructions (Life Science Cat. No. 11 667 165 001). To purify RNA from residual solvent and to remove any potential DNA contamination, RNA was subjected to a cleaning and DNAse treatment step using a kit from Zymo Research_(R1080). RNA concentrations were measured using a Nano DropTM 1000 spectrophotometer (Thermo Scientific), and the integrity/quality of the RNA was evaluated by capillary electrophoresis. To identify deregulated genes associated with apoptosis, autophagy, necroptosis, and oxidative stress, we used quantitative RT-PCR arrays (Human Cell Death Pathway 96 StellARray™ qPCR Array) from Qiagen. The list of genes is available online at https://www.qiagen.com/mx/products. Cell Stress Gene Array and Custom RT2 Profiler PCR Arrays (CAPH1376) were also purchased from Qiagen (Venlo, The Netherlands). Briefly, RNA was reverted to cDNA using M-MLV reverse transcriptase (QuantaBio, 95048), and 500 ng of cDNA was mixed with SYBR Green reagent (Thermo Fisher, 4368708). A total of 20 µL of the obtained mixture was applied to a 96-microarray plate, and quantification was performed in the StepOnePlus™ Real-Time PCR System (Applied Biosystems). A list of down- and upregulated genes was obtained by a comparative analysis of CT values from untreated and treated conditions using 2^−ddCt^.

### 4.8. Protein Extraction and Immunoblotting

Cells were seeded in 72 cm^2^ at a density of 0.8 × 10^6^ cells/mL. After treatment with the indicated concentrations of the specified drugs for the indicated time points, cells were harvested and washed twice with PBS. Total protein extraction was performed by using the M-PER Mammalian Extraction Reagent from Thermo Fisher (78505) supplemented phosphatase/protease inhibitor cocktail from Thermo Fisher (78440). The protein concentration was determined by BCA Protein Assay (23252). Western blotting was performed as described previously [36]. Caspase-3 (#9668), caspase-9 (#9508), CHOP (#2895), p-AKT (#4060), and PI3K antibodies were purchased from Cell Signaling.

### 4.9. Statistical Analysis

For statistical comparison of the flow cytometric and gene expression array analyses, a two-way ANOVA with Bonferroni post-hoc test for multiple comparisons was performed. A *p*-value less than or equal to 0.05 was considered statistically significant (Prism v5.0d, GraphPad Software, San Diego, CA, USA).

## Data Availability

The data presented in this study might be available depending on the type of the demand and the use, and are linked to the authorities’ authorization. A request must be sent to the corresponding author with the permission of all authors.

## Sample Availability

Samples of the compounds might be available depending on the type of the demand and the use, and are linked to the authorities’ authorization. A request must be sent to the corresponding author with the permission of all authors.

## Abbreviations

Allo-SCT	Allogeneic stem cell transplantation
AML	Acute myeloid leukemia
APC-annexin	Allophycocyanin annexin V
BCL-2	B-cell lymphoma 2
Car	Carvacrol
Casp-3	Caspase3
Casp-9	Caspase 9
Chop	C/EBP-homologous protein
DCFH-DA	Dichlorofluorescin diacetate
DMSO	Dimethylsulfoxide
DNA	Deoxyribonucleic acid
EO	Essential oil
ER	Endoplasmic reticulum
FBS	Fetal bovine serum
FLT3	FMS-like tyrosine kinase 3
HBSS	Hank’s Buffered Saline Solution
IGFR1	Insulin growth factor 1 receptor
MSC	Mesenchymal stromal cells
mToR	mammalian target of rapamycin
NAC	*N*-acetyl-l-cysteine
PBMC	Peripheral blood mononuclear cells
PBS	Phosphate-buffered saline
PI	Propidium iodide
PI3k	Phosphatidylinositol 3-kinase
PS	Phosphatidylserine
PV	*Ptychotis verticillata*
ROS	Reactive oxygen species
Thy	Thymol
UPR	Unfolded protein response
7-AAD	7-aminoactinomycine D
